# Sea Cucumber Glycosides: Chemical Structures, Producing Species and Important Biological Properties

**DOI:** 10.3390/md15100317

**Published:** 2017-10-17

**Authors:** Muhammad Abdul Mojid Mondol, Hee Jae Shin, M. Aminur Rahman, Mohamad Tofazzal Islam

**Affiliations:** 1 School of Science and Technology, Bangladesh Open University, Board Bazar, Gazipur 1705, Bangladesh; drmojidmondol@gmail.com; 2 Marine Natural Products Laboratory, Korea Institute of Ocean Science and Technology, 787 Haeanro, Ansan 427-744, Korea; 3 World Fisheries University Pilot Programme, Pukyong National University (PKNU), 45 Yongso-ro, Nam-gu, Busan 48513, Korea; aminur1963@gmail.com; 4 Department of Biotechnology, Bangabandhu Sheikh Mujibur Rahman Agricultural University, Gazipur 1706, Bangladesh

**Keywords:** holostane, nonholostane, cucumarioside, cytotoxic, antifungal, glycosides

## Abstract

Sea cucumbers belonging to echinoderm are traditionally used as tonic food in China and other Asian countries. They produce abundant biologically active triterpene glycosides. More than 300 triterpene glycosides have been isolated and characterized from various species of sea cucumbers, which are classified as holostane and nonholostane depending on the presence or absence of a specific structural unit γ(18,20)-lactone in the aglycone. Triterpene glycosides contain a carbohydrate chain up to six monosaccharide units mainly consisting of d-xylose, 3-*O*-methy-d-xylose, d-glucose, 3-*O*-methyl-d-glucose, and d-quinovose. Cytotoxicity is the common biological property of triterpene glycosides isolated from sea cucumbers. Besides cytotoxicity, triterpene glycosides also exhibit antifungal, antiviral and hemolytic activities. This review updates and summarizes our understanding on diverse chemical structures of triterpene glycosides from various species of sea cucumbers and their important biological activities. Mechanisms of action and structural–activity relationships (SARs) of sea cucumber glycosides are also discussed briefly.

## 1. Introduction

Nature is the largest source of pharmaceutical lead drugs for the remedies of many diseases. Earlier scientists mainly focused on terrestrial samples (plants and microorganisms) for the discovery of lead bioactive compounds. With the passage of time, the search for new drugs or agrochemicals has been switching from land to ocean due to re-isolation of known natural products from terrestrial samples. Marine organisms produce diversified bioactive compounds because of large species biodiversities and living in extremely harsh environment.

Among so many sources, numerous bioactive metabolites have been isolated from marine invertebrates such as echinoderms with a broad spectrum of biological activities [1]. The echinoderms are divided into five classes, i.e., Holothuroidea (sea cucumbers), Asteroidea (starfishes), Echinoidea (sea urchins), Crinoidea (sea lilies), and Ophiuroidea (brittle stars and basket stars), which live exclusively in the marine habitat, distributed in almost all depths and latitudes, as well as reef environments or shallow shores [2,3]. The importance of these echinoderms as a potential source of bioactive compounds for the development of new therapeutic drugs/agrochemicals has been growing rapidly [1]. Compounds isolated from echinoderms showed numerous biological activities including antibacterial, anticoagulant, antifungal, antimalarial, antiprotozoal, anti-tuberculosis, anti-inflammatory, antitumor, and antiviral activities [1]. 

Sea cucumber traditionally has been used as tonic food in China and other Asian countries for thousands of years. Besides being used as food, sea cucumbers are also promising source of bioactive natural products which predominantly belong to triterpene glycosides exhibiting antifungal, cytotoxic, hemolytic, cytostatic, and immunomodulatory and antiviral activities [4]. Several monographs concerning the structures and biological properties of triterpene glycosides obtained from sea cucumbers have been published but not presented in a systematic way [5,6]. This report comprehensively reviews in depth structural features of sea cucumber glycosides with corresponding producing species. Important biological activities, mechanism of action, and structure–activity relationships (SARs) of the diverse glycosides produced by the different species of sea cucumber are also discussed briefly.

## 2. Taxonomy, Distribution and Nutritive Value of Sea Cucumbers

One of the predominant invertebrate lives in marine environment is sea cucumber, which belong to the class Holothuroidea under the phylum Echinodermata. Holothuroidea has been divided into three subclasses, Aspidochirotacea, Apodacea and Dendrochirotacea, and further into six orders, Apodida, Elasipodida, Aspidochirotida, Molpadida, Dendrochirotida and Dactylochirotida [7]. Majority of the harvestable species of sea cucumbers belong to three families, viz., Holothuriidae (genera *Holothuria* and *Bohadschia*), Stichopodidae (genera *Stichopus*, *Actinopyga*, *Thelenota*, *Parastichopus* and *Isostichopus*), and Cucumariidae (genus *Cucumaria*) [8]. 

Sea cucumbers are elongated tubular or flattened soft-bodied marine benthic invertebrates, typically with leathery skin, ranging in length from a few millimeters to a meter [9]. Holothuroids encompass 14,000 known species occur in most benthic marine habitats worldwide, in both temperate and tropical oceans, and from the intertidal zone to the deep sea, and are considered as the very important parts of oceanic ecosystem [10]. 

Economically, sea cucumbers are important in two reasons: first, some species produce triterpene glycosides that are interested to pharmaceutical companies finding their medical use and second, use as food item. About 70 species of sea cucumbers have been exploited worldwide; out of which 11 species have been found to be commercially important [11]. Sea cucumbers have been well recognized as a tonic and traditional remedy in Chinese and Malaysian literature for their effectiveness against hypertension, asthma, rheumatism, cuts and burns, impotency and constipation [12,13]. Nutritionally, sea cucumbers have an impressive profile of valuable nutrients such as vitamin A, vitamin B_1_ (thiamine),vitamin B_2_ (riboflavin), vitamin B_3_ (niacin), and minerals, especially calcium, magnesium, iron and zinc [14,15].

## 3. Extraction, Purification and Characterization

To extract glycosides, first sea cucumbers will be freeze dried, then cut into pieces and extracted twice with refluxing EtOH. The combined extracts will be concentrated under reduced temperature and the residue will be dissolved in H_2_O. Desalting will be carried out by passing this fraction through a Polychrom column (Teflon), eluting first the inorganic salts and crude polar impurities with H_2_O and then the glycosides fraction with 50% EtOH. The fraction will be sub-fractionated by silica gel column chromatography using suitable gradient solvent system. The glycosides from each sub-fraction can be purified by reverse phase HPLC developing suitable solvent system (MeOH-H_2_O).

Triterpene glycosides have two parts: carbohydrate and triterpene. The number of monosaccharide units present in the carbohydrate chain can be deduced by observing the number of anomeric carbons (~103 ppm) and protons (~5 ppm, d) resonances in ^13^C and ^1^H NMR spectra, respectively. The sequence of monosaccharide units in the carbohydrate chain can be established by the analysis of anomeric H/C correlations in the HMBC spectrum which can also be confirmed by NOE corrections between anomeric protons and MALDI-TOF mass spectroscopic data analysis. The position of attachment of glycone with aglycone can be confirmed by the HMBC experiment.

The presence of diverse types of monosaccharide units and their repetitions in the carbohydrate chain can be established by acid hydrolysis followed by GC-MS analysis of the corresponding aldononitrile peracetates [16]. The site of attachment of sulfate group at monosaccharide units can be determined by observing chemical shift of esterification carbon atoms. The chemical shifts of α (esterification) and β-carbons are shifted ~5 ppm downfield and ~2 ppm up field, respectively, compare to their corresponding nonsulfated derivatives.

The structure of the aglycone can be established based on its spectroscopic data (^1^H NMR, ^13^C NMR, COSY, HMBC, HSQC, and TOCSY) and by comparing with the literature data. Configuration can be determined by the analysis of NOE data, stable conformers, coupling constants and comparing chemical shifts of chiral centers with literature.

## 4. Structural Features of Triterpene Glycosides Isolated from Sea Cucumbers

**Figure 1:**
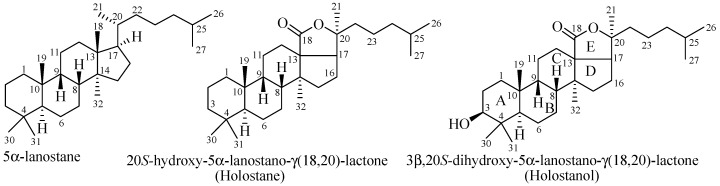
Structures of lanostane, holostane and holostanol.

Triterpene glycosides, also known as holothurins or saponins, are secondary metabolites typically produced by sea cucumbers (class Holothuroidea). These glycosides are amphiphilic in nature having two parts: aglycone (lipophilic, lipid-soluble) and glycone (hydrophilic, water-soluble). The majority of the glycosides contain so called holostane type aglycone comprise of lanostane-3β-ol with a γ(18,20)-lactone in the E-ring of the pentacyclic triterpene [(3β,20*S*-dihydroxy-5α-lanostano- γ(18,20)-lactone] (Figure 1). A few of the glycosides contain nonholostane type aglycone which do not have γ(18,20)-lactone in the tetracyclic triterpene.

**Figure 2:**
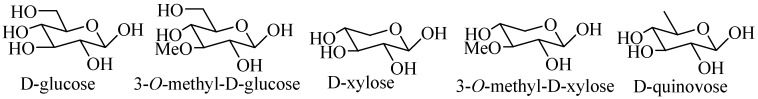
Common sugar units present in sea cucumber glycosides.

The glycone parts may contain up to six monosaccharide units covalently connected to C-3 of the aglycone. The sugar moieties mainly consist of d-xylose (Xyl), d-quinovose (Qui), d-glucose (Glc), 3-*O*-methyl-d-glucose (MeGlc), 3-*O*-methyl-d-xylose (MeXyl) (Figure 2) and sometimes 3-*O*-methyl-d-quinovose (MeQui), 3-*O*-methyl-d-glucuronic acid (MeGlcA) and 6-*O*-acetyl-d-glucose (AcGlc). In the carbohydrate chain, the first sugar unit is always a xylose and a majority case second is quinovose, whereas 3-*O*-methyl-d-glucose and/or 3-*O*-methyl-d-xylose are always the terminal monosaccharide units. The presence of two quinovose residues in a carbohydrate chain is unique for sea cucumber and starfish glycosides.

**Figure 3:**
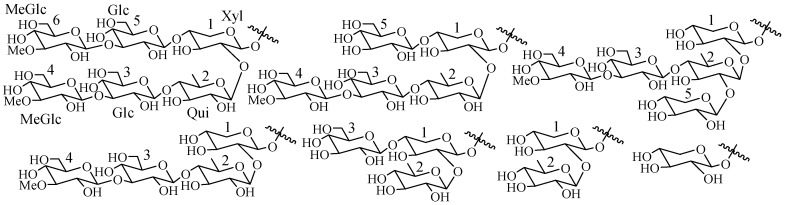
Some common carbohydrate architectures found in sea cucumber glycosides.

In glycone part, the sugar units are generally arranged in a straight or branched chain (Figure 3). The majority of tetrasaccharides show a linear chain with the most common 3-*O*-Me-Glc-(1→3)-Glc-(1→4)-Qui-(1→2)-Xyl. Hexaglycosides are generally nonsulfated with a linear 3-*O*-Me-Glc (1→3)-Glc (1→4)-Xyl (2→1)-Qui (4→1)-Glc (3→1)-3-*O*-MeGlc unit. Pentasaccharides have a linear chain like tetrasaccharides but a branching at C-2 of quinovose (Figure 3).

Sixty percent of the triterpene glycosides isolated so far from sea cucumbers have sulfate groups linked to the monosaccharide units of the carbohydrate chain. Most of them are monosulfsated, but many di- and trisulfated glycosides have also been isolated. Most tetrasaccharides and pentasaccharides are sulfated at C-4 of xylose unit. In both the cases, additional sulfate groups at C-6 of the 3-*O*-methylglucose and glucose units have also been found. The term “Ds” stands for desulfated. Sea cucumber triterpene glycosides are chemotaxonomic markers specific for groups of genera within each family.

Triterpene glycosides can be classified as holostane type having 3β-hydroxy-5α-lanostano- γ(18,20)-lactone structural feature and nonholostane type do not have a γ(18,20)-lactone but have other structural features like holostane type glycosides.

### 4.1. Holostane Type Triterpene Glycosides

**Figure 4:**
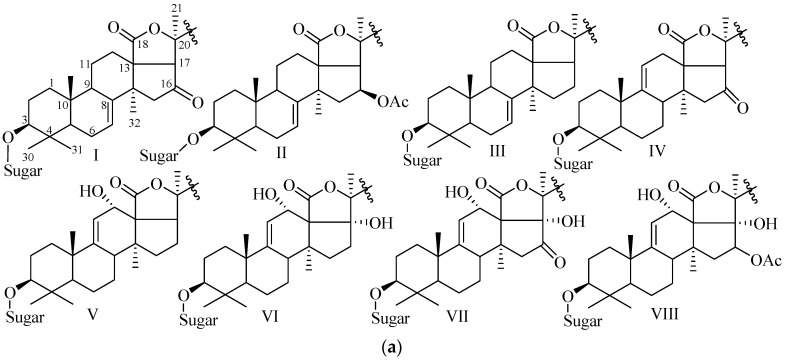

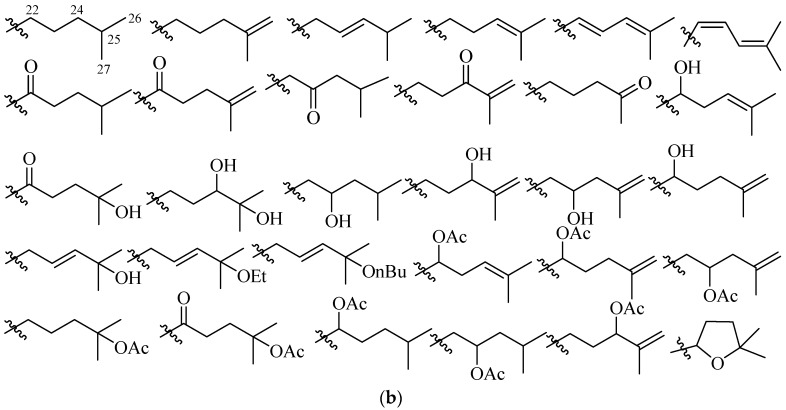
Pentacyclic triterpene and alkane side chain skeletons are commonly found in holostane type glycosides. (**a**) Pentacylic triterpene skeletons. Substitution by selective functional groups and unsaturation generally take place in the alkane side chain (2-methylpentane) attached to C-20 of the E-ring of aglycone; (**b**) Alkane side chain architectures.

Depending on the position of double bond in the B and C ring of the aglycone (Figure 1), holostane type glycosides can be further subdivided into three groups: glycosides with 3β-hydroxyholost-7(8)-ene, 3β-hydroxyholost-9(11)-ene, and 3β-hydroxyholost-8(9)-ene aglycone skeletons. There are eight pentacyclic triterpene and 30 alkane side chain aglycone architectures commonly found in holostane type glycosides (Figure 4). In these architectures, certain functional groups are generally attached to the specific carbons: keto and β-acetoxy groups at C-16, and α-hydroxy group at C-12 and C-17.

#### 4.1.1. 3β-Hydroxyholost-7(8)-ene Skeleton Containing Holostane Glycosides

Substantial number of triterpene glycosides in this category is produced by sea cucumbers. The species *Eupentacta fraudatrix, Holothuria lessoni, Bohadschia marmorata, Stichopus chloronotus* and *Staurocucumis liouvillei* produce most of the compounds in this group. For convenience, the large number of compounds in this category can be further subdivided into four groups depending on the number of sugar units.

##### Holostane Glycosides with 3β-Hydroxyholost-7(8)-ene Skeleton and Six Sugar Units

**Figure 5:**
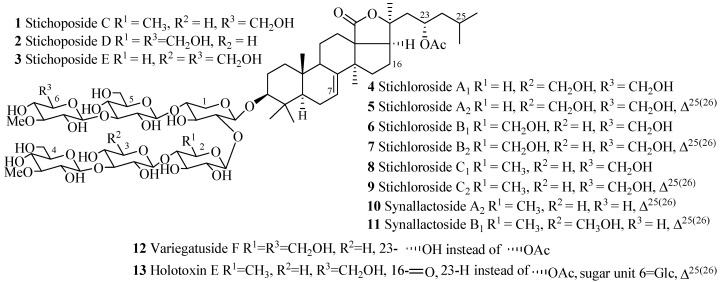
Chemical structures of holostane glycosides with 3β-hydroxyholost-7(8)-ene and six sugar units.

The name of the compounds in this group, their producing species, chemical structures and references are summarized in Table 1 and Figure 5. The most common features of glycosides in this category are the presence of α-acetoxy group at C-23, double bond at C-25(C-26) and terminal 3-*O*-methyl-d-glucose in carbohydrate chain. An interesting point to be noted in here is that the sulfate group is totally absent in this group of compounds.

##### Holostane Glycosides with 3β-Hydroxyholost-7(8)-ene Skeleton and Five Sugar Units

**Figure 6:**
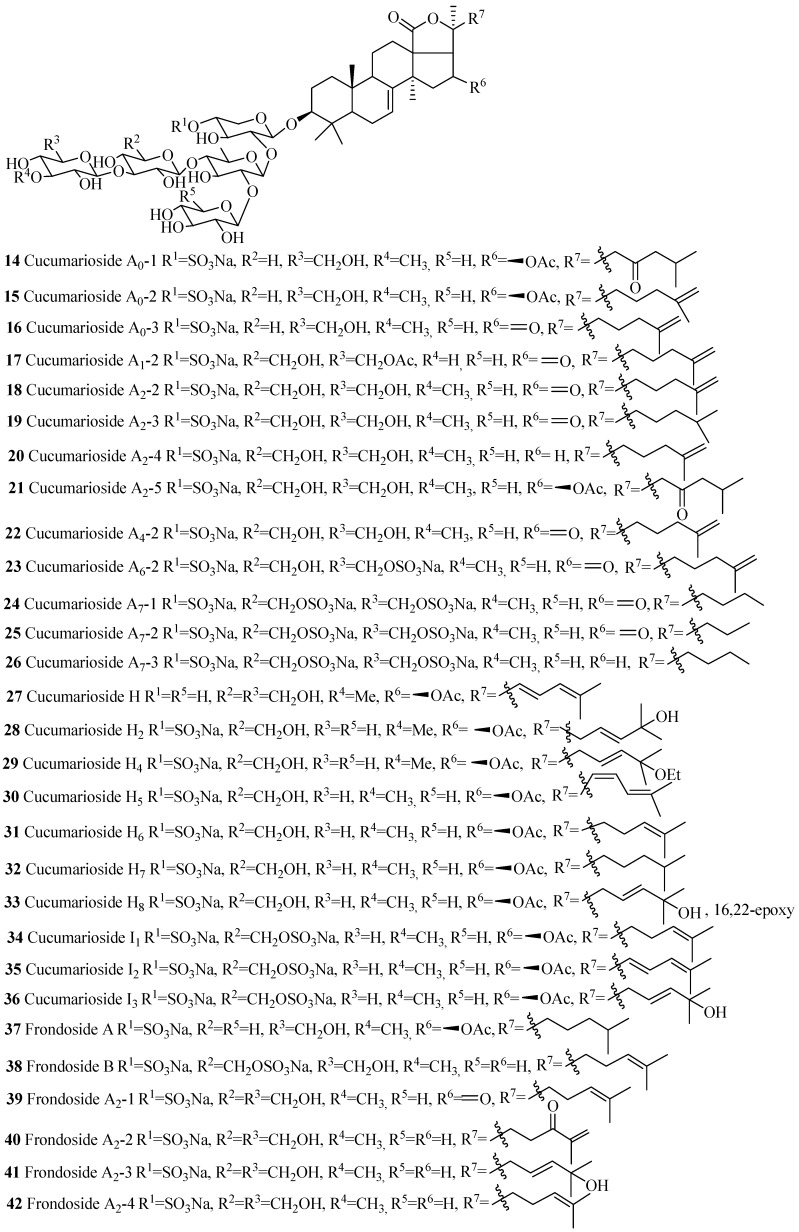

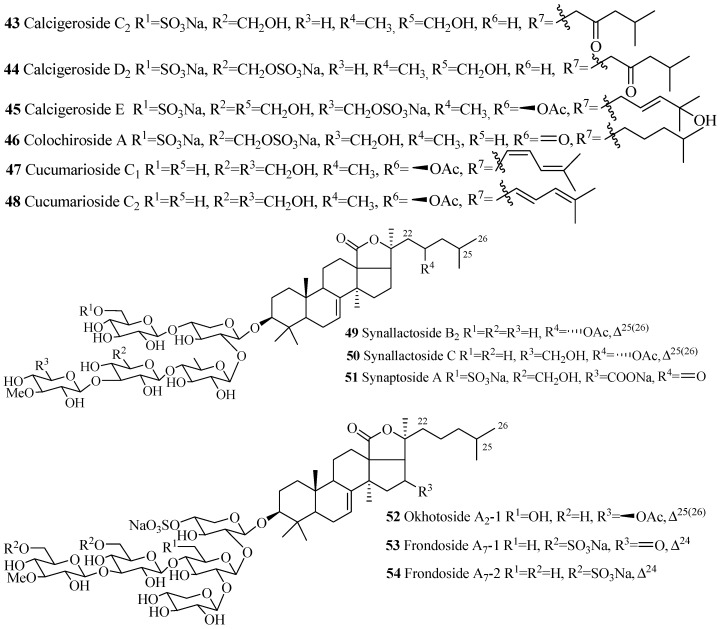
Chemical structures of holostane glycosides with 3β-hydroxyholost-7(8)-ene and five sugar units.

The name of the compounds in this group, their producing species, chemical structures and references are summarized in Table 2 and Figure 6. The most common structural features in this group are the sulfate groups at C-4 of xylose and C-6 of glucose and methylglucose with either β-acetoxy or keto group at C-16 and C-25(26) double bond. A quite number of compounds contain a keto group at C-23.The rare structural features of triterpene glycoside are the presence of 16,22-epoxy group (**33**), ethoxy group (**29**) and methylglucuronic acid (**51**). Cucumarioside A_1_-2 (**17**) is the only example of triterpene glycosides containing an acetate group at C-6 of the terminal sugar unit. Carbohydrate chain can be one branched (**14**–**48** and **52**–**54**) or straight (**49**–**51**). 3-*O*-methyl-d-xylose as a terminal monosaccharide unit that is a characteristic feature of all the glycosides isolated from *Eupentacta fraudatrix*.

##### Holostane Glycosides with 3β-Hydroxyholost-7(8)-ene Skeleton and Four Sugar Units

**Figure 7:**
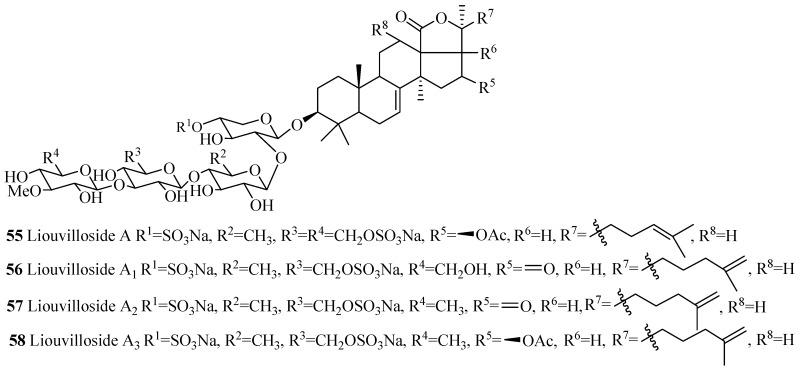

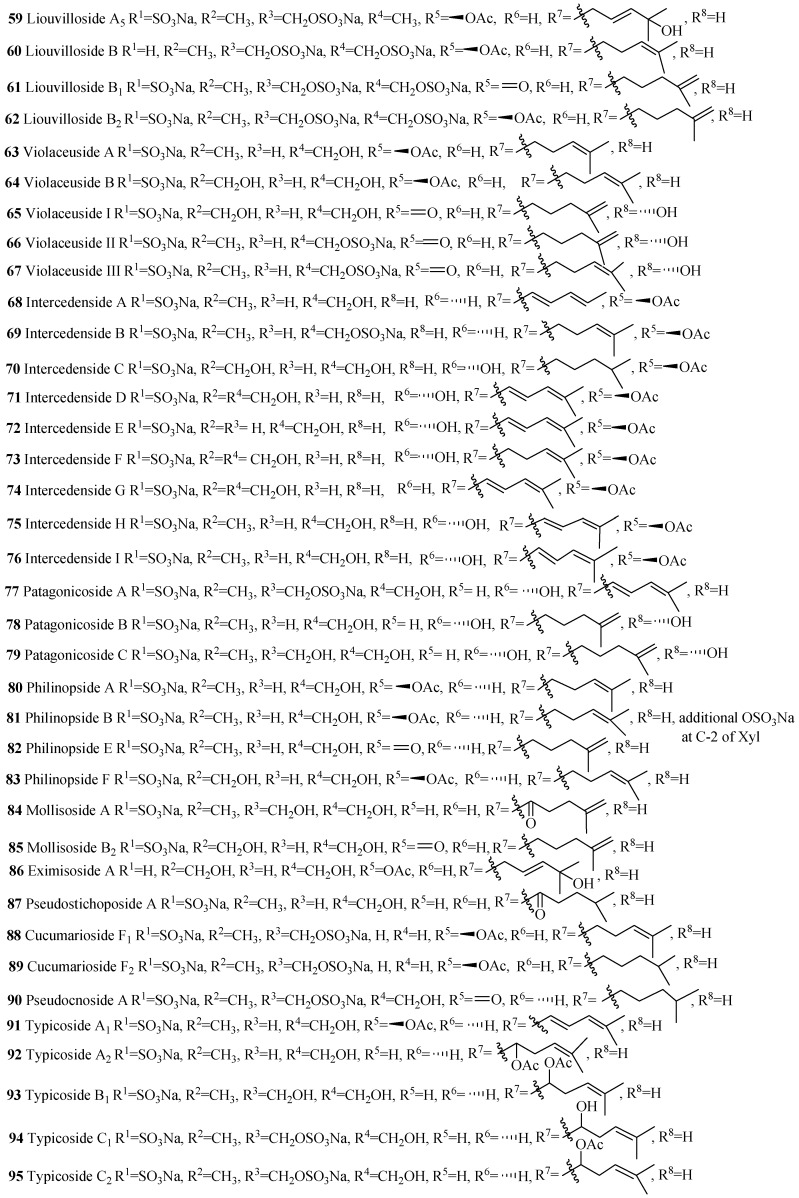

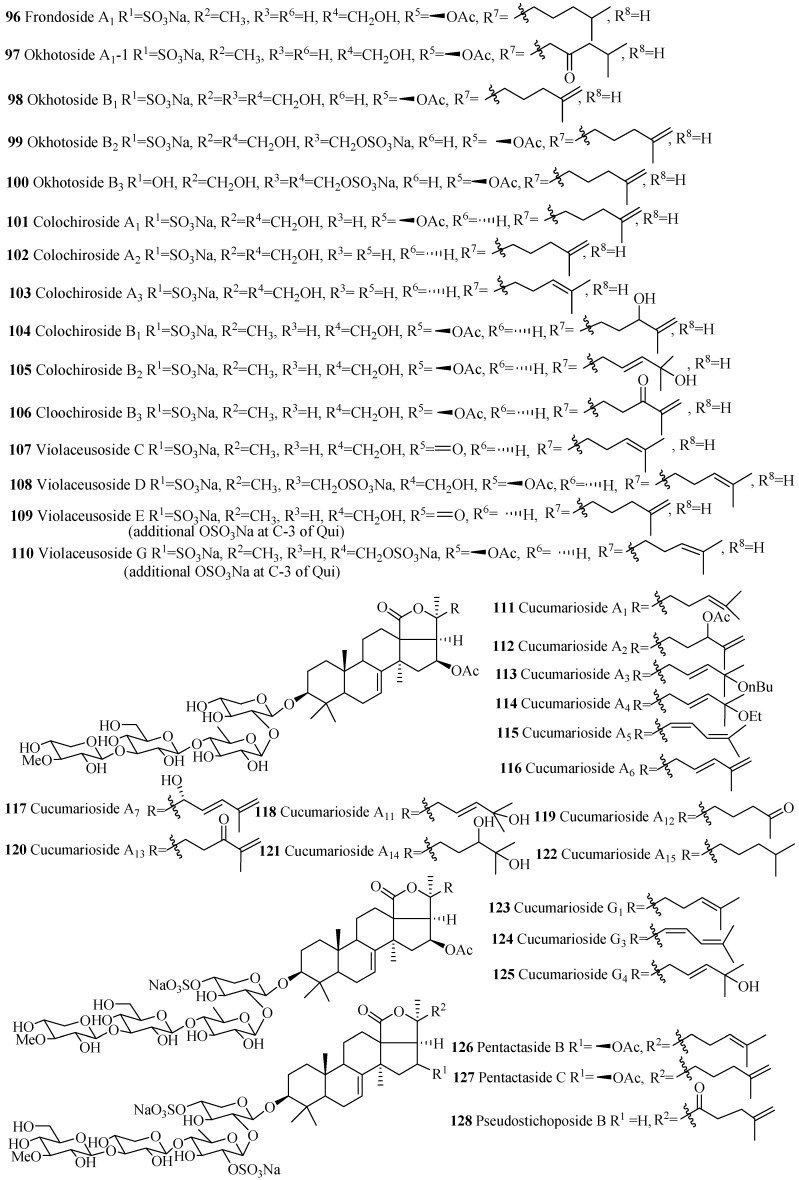

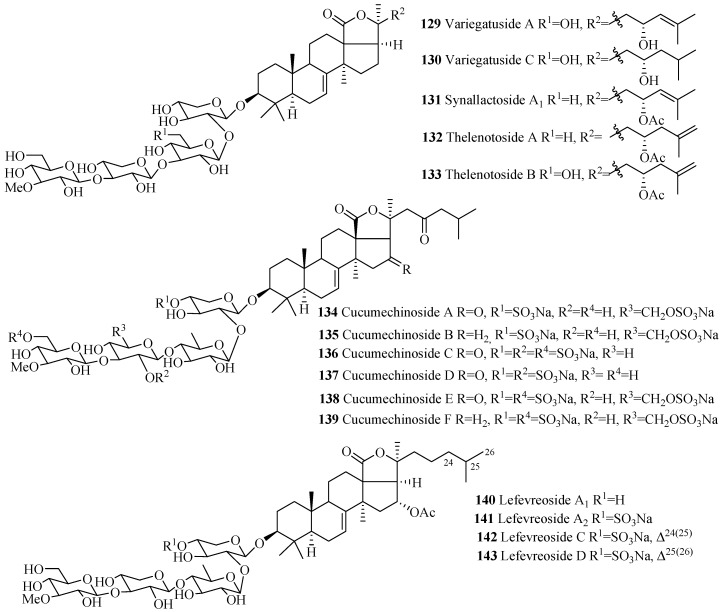
Chemical structures of holostane glycosides with 3β-hydroxyholost-7(8)-ene and four sugar units.

Several compounds in this group were isolated from the species of *Staurocucumis liouvillei* and *Eupentacta fraudatrix* (Table 3). The most common characteristic of glycosides in the group is the presence of sulfate at C-4 of xylose and either keto or β-acetoxy group at C-16 (Figure 7). Some of the compounds in this series, especially liouvillosides, violaceusosides and cucumechinosides, may contain up to three sulfates in their carbohydrate chain. The presence of *α*-hydroxy at C-12 and C-17 (**78** and **79**), artifact *n*-butoxy (**113**) and ethoxy (**114**) groups at C-25, and three consecutive xylose sugar units in carbohydrate chain (**72**) are rare structural features in this category. Cucumariosides A_1_ (**111**), A_5_ (**115**) and A_11_ (**118**) are the desulfated derivatives of cucumariosides G_1_ (**123**), G_3_ (**124**) and G_4_ (**125**), respectively. 

##### Holostane Glycosides with 3β-Hydroxyholost-7(8)-ene Skeleton and 1–3 Sugar Units

**Figure 8:**
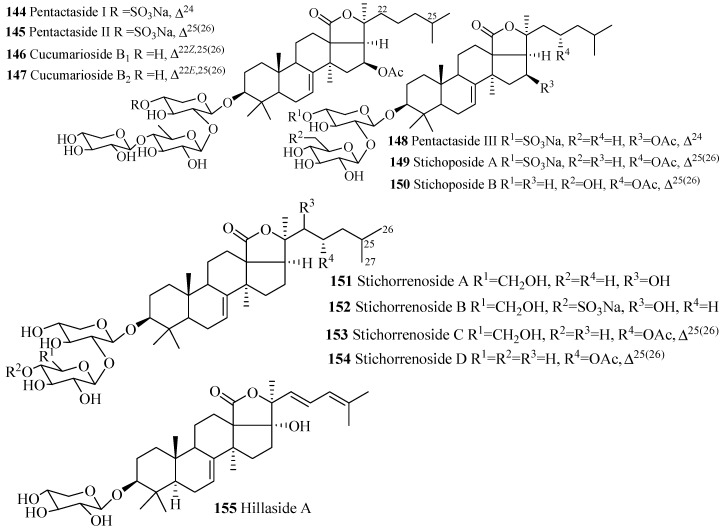
Chemical structures of holostane glycosides with 3β-hydroxyholost-7(8)-ene and 1–3 sugar units.

The name of the compounds in this group, their producing species, chemical structures and references are summarized in Table 4 and Figure 8. The most common feature of triterpene glycosides is the presence of double bond at C-25(26). Cucumarioside B_1_ (**146**) is the geometric isomer of cucumarioside B_2_ (**142**) and pentactaside III (**148**) is the positional isomer of stichoposide A (**153**).

#### 4.1.2. 3β-Hydroxyholost-9(11)-ene Skeleton Containing Holostane Glycosides

The species *Holothuria lessoni, Bohadschia marmorata and Bohadschia bivittata* produce most of the compounds in this group. For convenience, the large number of compounds in this category can also be further subdivided into four groups depending on the number of sugar units

##### Holostane Glycosides with 3β-Hydroxyholost-9(11)-ene Skeleton and Six Sugar Units

**Figure 9:**
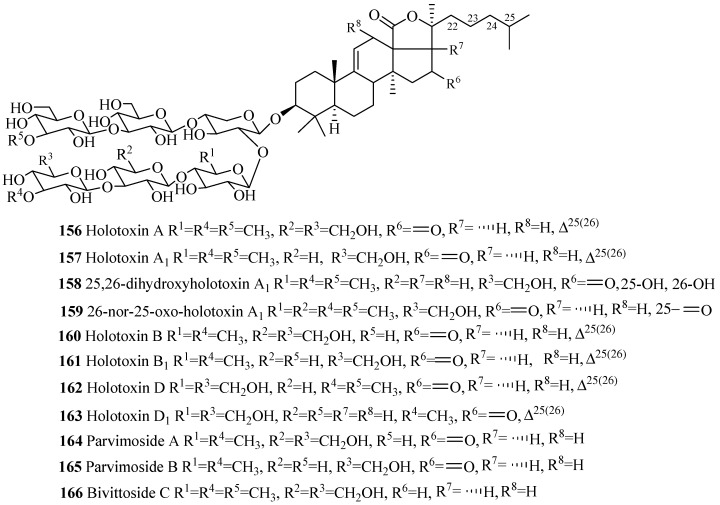

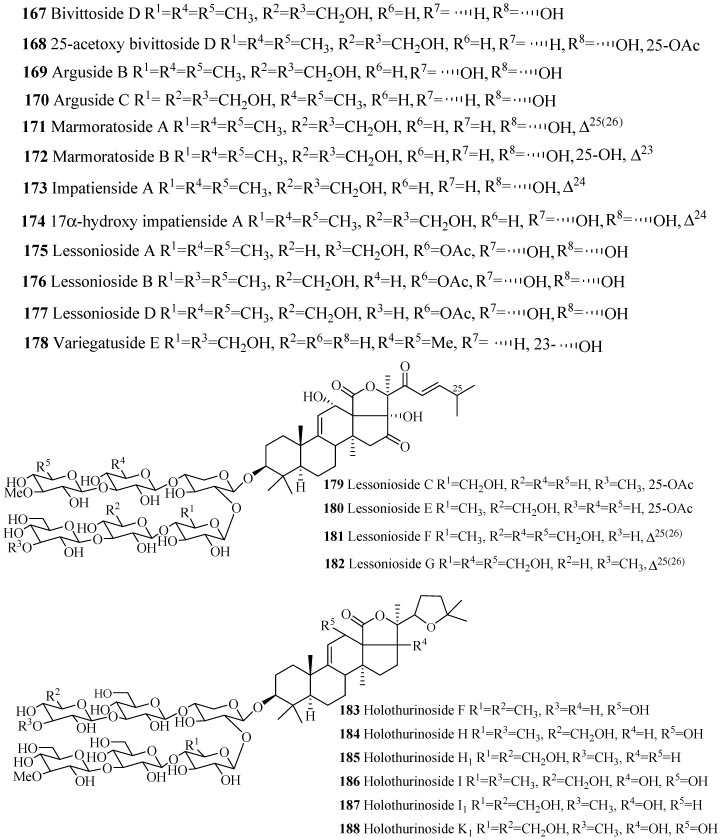

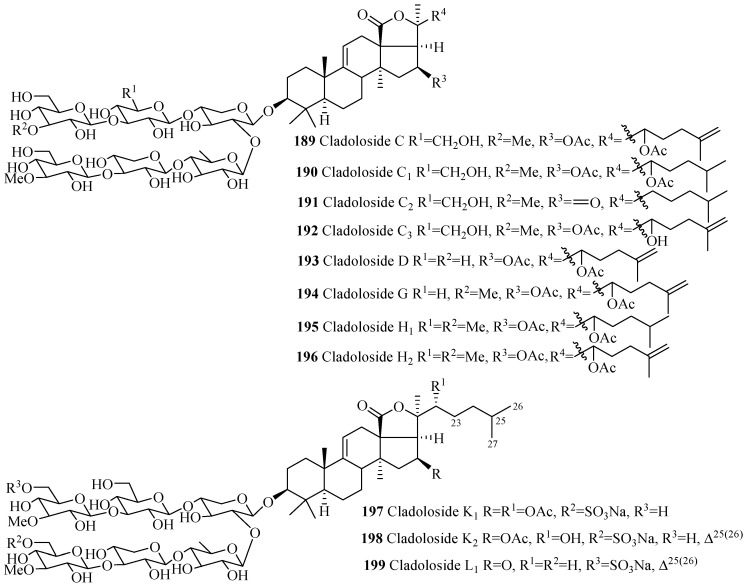
Chemical structures of holostane glycosides with 3β-hydroxyholost-9(11)-ene and six sugar units.

Similar to 3β-hydroxyholost-7(8)-ene skeleton with six sugar units (Figure 5), 3β-hydroxyholost-9(11)-ene skeleton with six sugar units glycosides also do not have any sulfate group in their carbohydrate chain (Figure 9 and Table 5) except cladolosides K_1_, K_2_ and L_1_ (**197**–**199**). The most common structural feature of triterpene glycosides in this category is the presence of 3-*O*-methyl-d-glucose at the both end of the straight carbohydrate chain. Holotoxin and parvimoside series (**156**–**166**) of compounds have a keto group at position C-16. Double bond at C-25(26) among holotoxins (**156**–**163**) is common, except 26-nor-25-oxo-holotoxin A_1_ (**159**), where the double bond is replaced by a keto group. The α-hydroxy groups at C-12 and C-17 are commonly found in the aglycone part of lessonioside series of glycosides (**175**–**177**). The α-hydroxy group at C-12 and C-17, and 22,25-epoxy are common structural characteristics of holothurinosides (**183**–**188**). Acetoxy group at C-16 and C-22 are frequently observed in cladoloside glycosides (**189**–**199**).

##### Holostane Glycosides with 3β-Hydroxyholost-9(11)-ene Skeleton and Five Sugar Units

**Figure 10:**
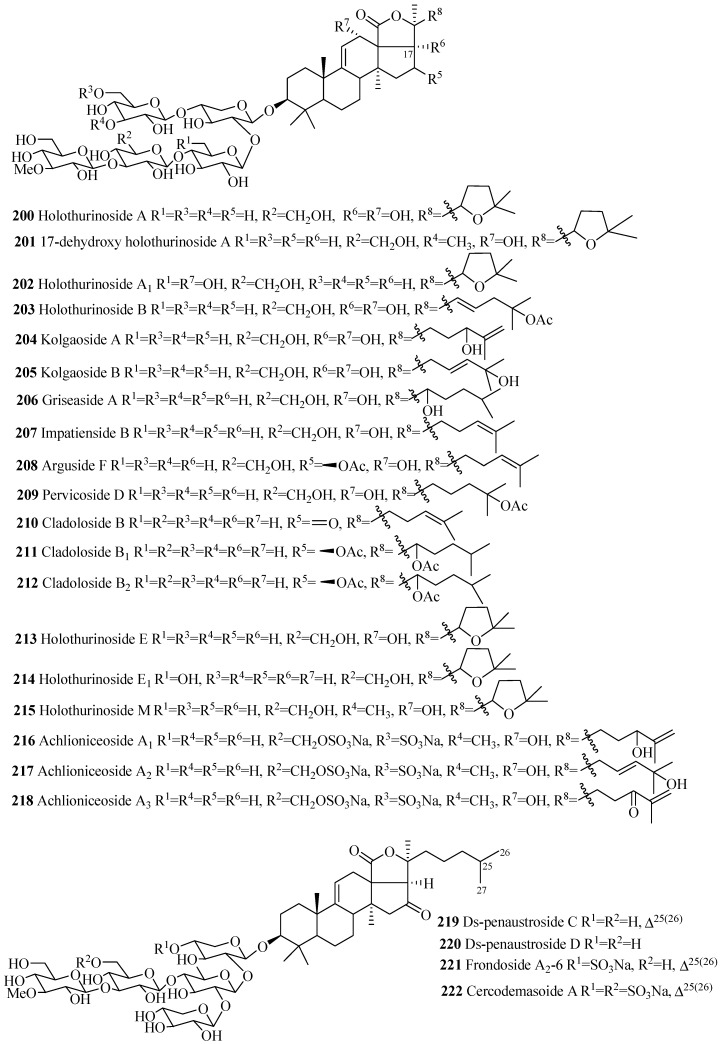

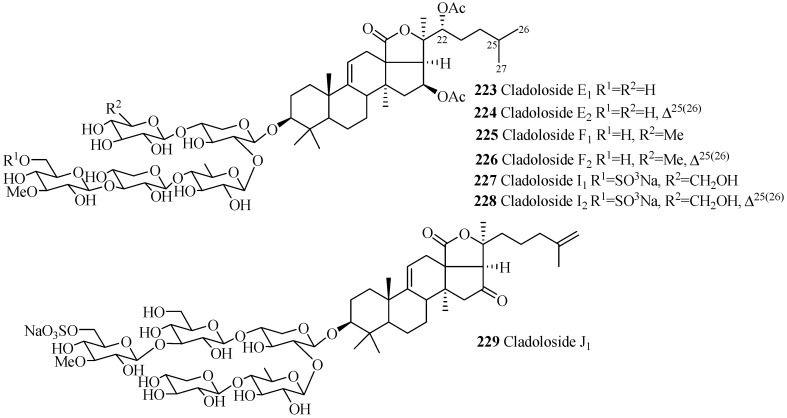
Chemical structures of holostane glycosides with 3β-hydroxyholost-9(11)-ene and five sugar units.

The carbohydrate chains of glycosides in this group are either straight (**200**–**218** and **223**–**229**) or branched (**219**–**222**) (Figure 10 and Table 6). The 22,25-epoxy (**200**–**202**, **213**–**215**) and two acetoxy groups, one at C-16 and another at C-22 (**211**, **212**, **223**–**228**), are common in holothurinosides and cladolosides, respectively. Kolgaosides (**204** and **205**) and achlioniceosides (**216**–**218**) within their own groups have the same carbohydrate chains and the only difference is in their respective aglycone side chains. 

##### Holostane Glycosides with 3β-Hydroxyholost-9(11)-ene Skeleton and Four Sugar Units

**Figure 11:**
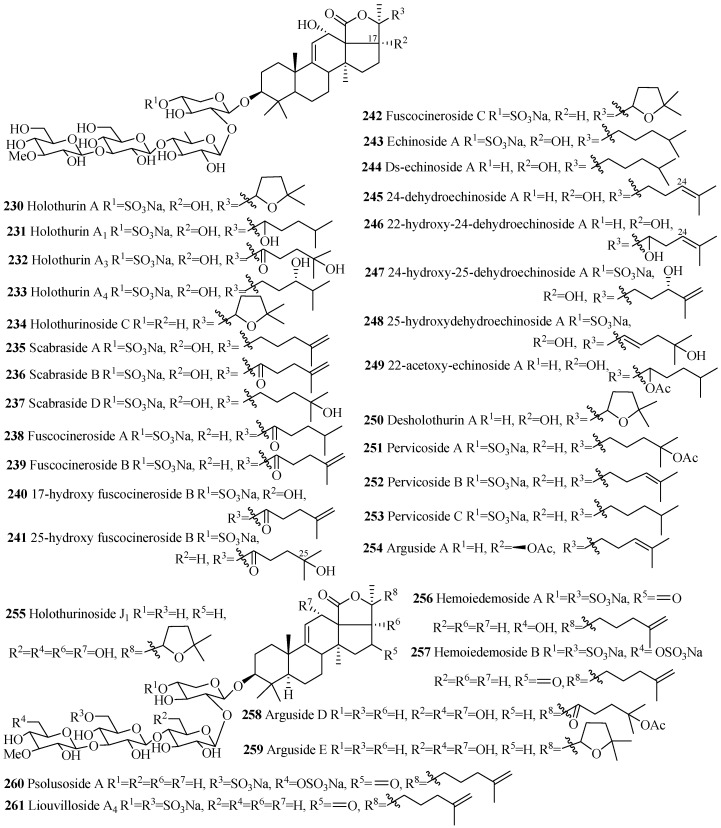

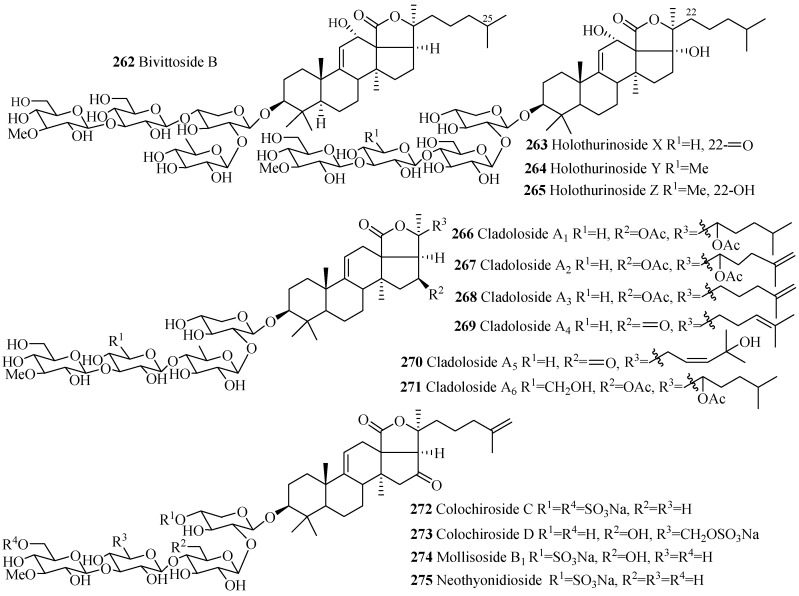
Chemical structures of holostane glycosides with 3β-hydroxyholost-9(11)-ene and fours ugar units.

The names and structures of the glycosides belonging to this group are summarized in the Table 7 and Figure 11. Almost all the saponins in this group contain sulfate group at C-4 of xylose sugar. The most common features of holothurins (**230**–**233**), scabrasides (**235**–**237**) and echinosides (**243**–**249**) are the presence of hydroxy groups at C-12 and C-17 (Figure 11). Among the cladoloside series of compounds (**266**–**271**), either keto or acetoxy group is commonly found at position C-16 and 22. The uncommon linear sugar chain [3-*O*-MeGlc (1→3)-Glc (1→4)-Xyl (2→1)-Qui] is observed in bivittoside B (**262**). Another exceptional feature has been found in this category of compounds is the presence of three consecutive glucose unit in the linear carbohydrate chain (**258** and **259**). 

##### Holostane Glycosides with 3β-Hydroxyholost-9(11)-ene Skeleton and 1–3 Sugar Units

**Figure 12:**
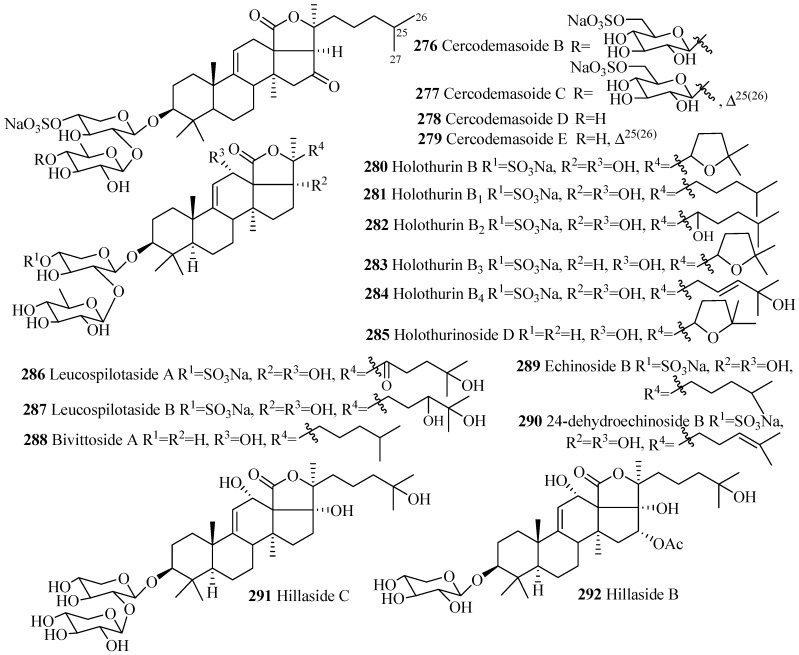
Chemical structure of holostane glycosides with 3β-hydroxyholost-9(11)-ene and 1–3 sugar units.

Only one type of carbohydrate chain, d-xylose-d-quinovose, is found in all glycosides in this group having two monosaccharide units (**278**–**290**), except **291** where carbohydrate chain is d-xylose-d-xylose (Figure 12 and Table 8); sulfate groups at C-4 of xylose units are also commonly found as well, except **285**, **288** and **291**. Hydroxy groups at either C-12 or C-17, or both positions, are observed in all the compounds in this category (Figure 12), except cercodemasoides (**276**–**279**).

#### 4.1.3. Holostane Glycosides with 3β-Hydroxyholost-8(9)-ene Skeleton

**Figure 13:**
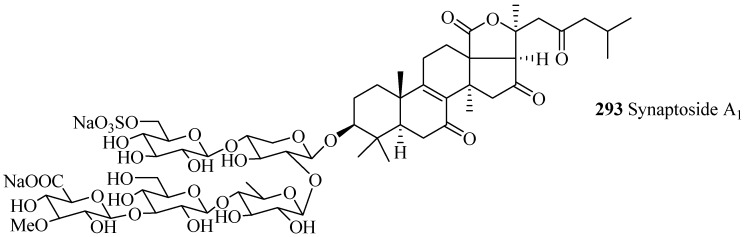

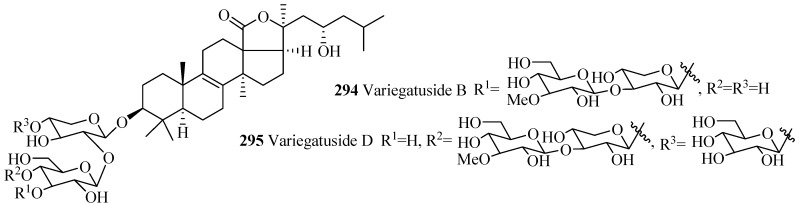
Chemical structures of holostane glycosides with 3β-hydroxyholost-8(9)-ene skeleton.

Only three glycosides belong to this group with carbohydrate chain consisting of 4–5 monosaccharide units (Table 9 and Figure 13). Among holostane sea cucumber glycosides, only one glycoside, synaptoside A_1_ (**293**), contains keto group at C-7. 

### 4.2. Nonholostane Glycosides

**Figure 14:**
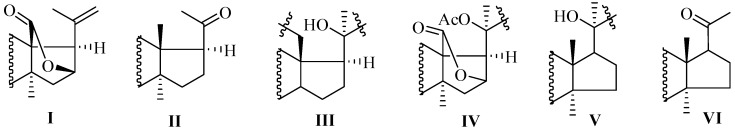
D- and E-ring structural architectures present in nonholotane glycosides.

**Figure 15:**
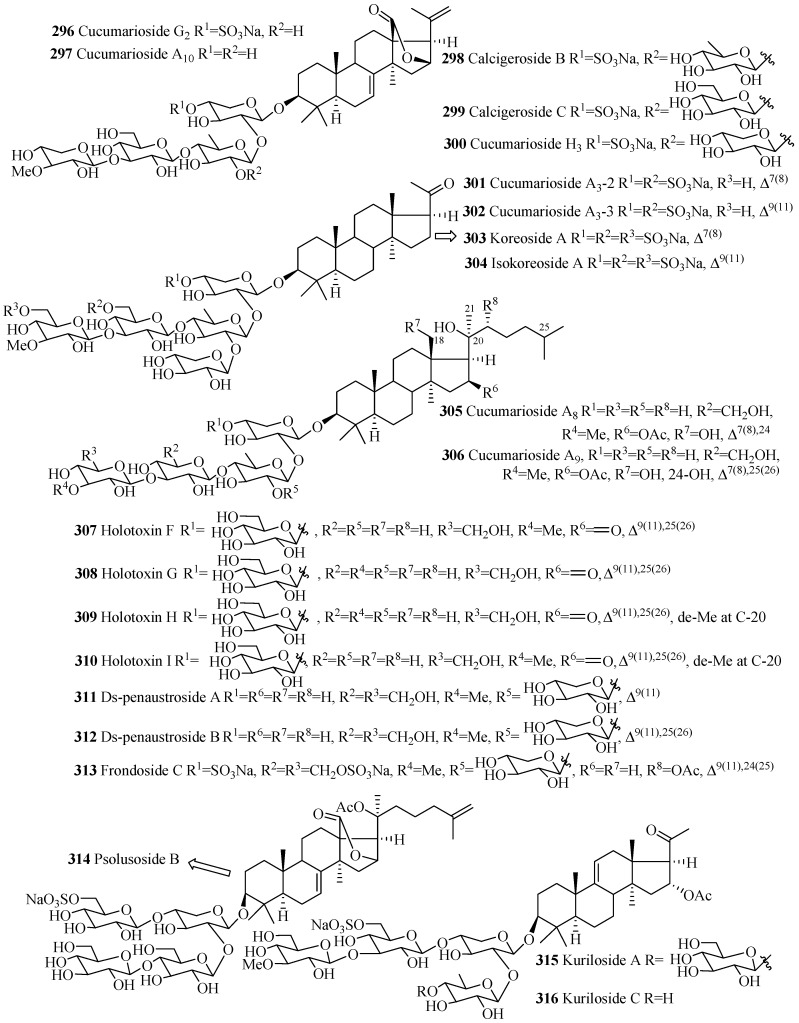

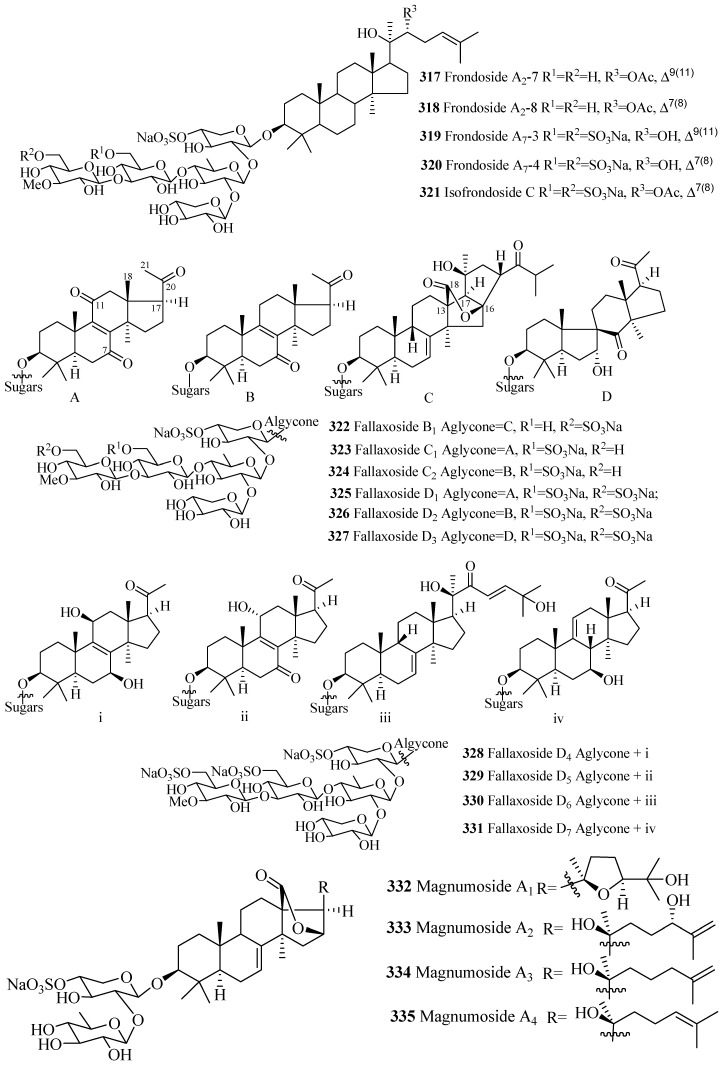

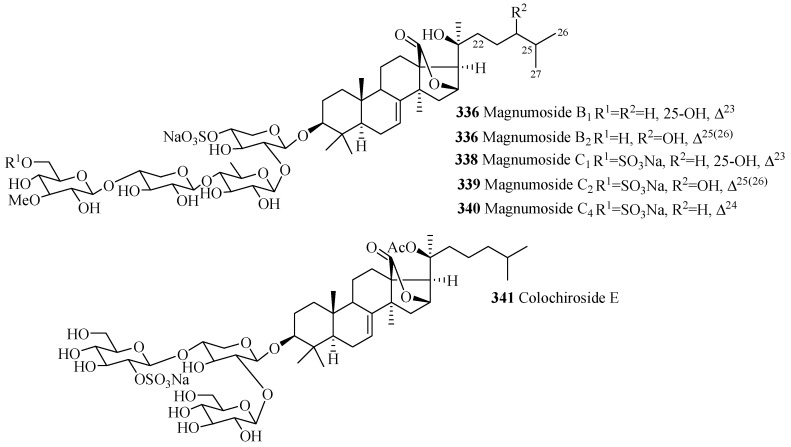
Chemical structures of nonholostane glycosides.

As mention earlier, like holostane glycosides, nonholostane glycosides do not have γ(18,20)-lactone structural unit (Figure 14 and Figure 15, Table 10). There are six different structural units (Figure 14) present in D- and E rings of aglycone in nonholostane glycosides. The aglycone side chain can be long or short, and may contain keto, methylene, hydroxy and acetoxy functional groups (Figure 15). Instead of γ(18,20)-lactone, some glycosides in this group contain γ(16,18)-lactone (**296**–**300**, **314**, **322**, **332**–**340** and **341**). Cucumariosides A_8_ (**305**) and A_9_ (**306**) contain uncommon hydroxy group at C-18. Fallaxosides B_1_ (**322**) and D_3_ (**327**) are novel glycosides with unprecedented skeletons of aglycones. Psolusoside B (**314**) and Kuriloside C (**316**) have four members sugar architecture which are uncommon in both holostane and nonholostane glycosides. Another uncommon feature of this group of compounds is the presence of keto group at C-11 (**323** and **325**). Sulfate group is commonly found at C-4 of first xylose unit (Figure 15). Most of the nonholostane glycosides have branched five members carbohydrate chain (Figure 15). 

## 5. The Important Biological Properties of Sea Cucumber Glycosides

Triterpene glycosides are the prime bioactive metabolites of sea cucumbers, and are commonly known as toxins of sea cucumbers to eukaryotic cells. These glycosides showed a wide range of biological activities including cytotoxic, antifungal, antiviral, hemolytic, antiprotozoal and immunomodulatory activities. Sea cucumbers produce some major glycosides in sufficient amount to carry out a wide range of biological activity tests [37,94]. Besides major glycosides, they also produce minor glycosides insufficient to test a range of biological activities [66,67]. The point to be noted here is that sea cucumber glycosides are able to exhibit biological activities in both in vitro and in vivo models [5]. The remarkable biological properties showed by some triterpene glycosides are summarized in Table 11. Triterpene glycosides do not exhibit antibacterial activity, indicating that these glycosides are probably produced by sea cucumbers for defence against eukaryotic predators.

## 6. Mechanisms of Action

Natural products derived from marine organisms have incredible structural and functional diversity. The mechanism by which triterpene glycosides exhibit anticancer activity primarily involve induction of tumor cell apoptosis through the activation of intracellular caspase cell death pathways, arrest of the cell cycle at S or G_2_/M phases and increase of the sub-G_0_/G_1_ cell population; regulation of nuclear factor NF-κB expression; reduction in cancer cell adhesion; suppression of cell migration and tube formation; suppression of angiogenesis; inhibition of cell proliferation, colony formation, and tumor invasion [141]. However, the detailed mechanism(s) of the anticancer activities of these glycosides remains largely unclear.

Marked membranolytic effects such as increased membrane permeability, loss of barrier function, and the rupture of cell membrane are considered the basic mechanisms underlying a variety of biological activities exerted by triterpene glycosides of sea cucumbers. The glycosides form complex with ∆^5(6)^-sterols of cellular membrane especially cholesterol. This interaction induces significant changes in the physicochemical properties of cell membranes, such as variations in their stability, microviscosity, and permeability. Saponins form complexes with membrane sterols, leading to cell disruption by the formation of pores. Due to this irreversible interaction, the selective permeability of cell membranes is impaired and cell compounds are transferred into the extracellular matrix, ultimately resulting in cell death [142,143].

## 7. Structure–Activity Relationships (SARs)

Both glycone and aglycone parts are important for biological activities of sea cucumber glycosides. The structure–activity relationships among sea cucumber glycosides are presumably more complicated. The most important structural characteristics of glycosides that probably contribute in biological activities are mentioned below.

The cytotoxicity not only depends on the chemical structures of the glycosides but also cell types [144]. The presence of 12α-hydroxy and 9(11)-ene structural units in holostane aglycone play key role in cytotoxicity [144]. Number of monosaccharide units in sugar chains and the substitution in side chain of aglycone could affect cytotoxicity. The presence of hydroxy groups in the side chains of glycosides significantly reduces cytotoxicity of the glycosides with increasing distance of hydroxy group from the 18(20)-lactone [30,31]. Linear tetraoside unit plays important role in different biological activities of sea cucumber glycosides [144]. Hexaoside chain containing glycosides show stronger cytotoxic activity than pentaoside chain containing glycosides. Glycosides with hexaosides residue with xylose or quinovose in the fifth position are the most active cytotoxins [144]. Different activities test result indicates that the number of sulfate groups and their position in the carbohydrate chains affect cytotoxicity [144]. It has been shown that the sulfate group attached to C-6 of terminal 3-*O*-methylglucose unit greatly decrease and attached to C-6 of glucose (the third monosaccharide unit) generally increase membranotropic activity [145].

## 8. Conclusions

Sea cucumbers (or holothurians), a class of marine invertebrates, are used as human food and traditional medicine, especially in some parts of Asia. The majority of the sea cucumbers synthesize glycosides with a polycyclic aglycone that contain either 7(8)- or 9(11)-double bond with up to six monosaccharide residues containing carbohydrate chain. A few of them are known to synthesize aglycone with 8(9)-ene. Sea cucumber glycosides are cytotoxic to eukaryotes; probably produce for escaping from predation by marine eukaryotic organisms. These cucumber metabolites have shown profound cytotoxic and hemolytic activities against eukaryotic organisms but not prokaryotic organisms. Due to significant cytotoxic and antifungal activities, extensive differential SAR studies of these glycosides can be helpful to develop new drugs and agrochemicals.

## Figures and Tables

**Table 1:** Name and producing species of glycosides with 3β-hydroxyholost-7(8)-ene and sixs ugar units.

Compound Name	Producing Species	Reference	Compound Name	Producing Species	Reference
Stichoposide C (**1**)	*Thelenota anax*	[17]	Stichoposide D (**2**)	*Thelenota anax*	[18]
Stichoposide E (**3**)	*Stichopus chloronotus*	[19]	Stichloroside A_1_ (**4**)	*S. chloronotus*	[20]
Stichloroside A_2_ (**5**)	*S. chloronotus*	[20]	Stichloroside B_1_ (**6**)	*S. chloronotus*	[20]
Stichloroside B_2_ (**7**)	*S. chloronotus*	[20]	Stichloroside C_1_ (**8**)	*S. chloronotus*	[20]
Stichloroside C_2_ (**9**)	*S. chloronotus*	[20]	Synallactoside A_2_ (**10**)	*Synallactes nozawai*	[16]
Synallactoside B_1_ (**11**)	*S. nozawai*	[16]	Variegatuside F (**12**)	*S. variegates*	[21]
Holotoxin E (**13**)	*S. japonicus*	[22]			

**Table 2:** Name and producing species of glycosides with 3β-hydroxyholost-7(8)-ene and five sugar units.

Compound Name	Producing Species	Reference	Compound Name	Producing Species	Reference
Cucumarioside A_0_-1 (**14**)	*Cucumaria japonica*	[23]	Cucumarioside A_0_-2 (**15**)	*C. japonica*	[23]
Cucumarioside A_0_-3 (**16**)	*C. japonica*	[23]	Cucumarioside A_1_-2 (**17**)	*C. japonica*	[24]
Cucumarioside A_2_-2 (**18**)	*C. japonica*	[25]	Cucumarioside A_2_-3 (**19**)	*C. japonica*	[24]
Cucumarioside A_2_-4 (**20**)	*C. japonica*	[24]	Cucumarioside A_2_-5 (**21**)	*C. conicospermium*	[26]
Cucumarioside A_4_-2 (**22**)	*C. japonica*	[24]	Cucumarioside A_6_-2 (**23**)	*C. japonica*	[27]
Cucumarioside A_7_-1 (**24**)	*C. japonica*	[28]	Cucumarioside A_7_-2 (**25**)	*C. japonica*	[28]
Cucumarioside A_7_-3 (**26**)	*C. japonica*	[28]	Cucumarioside H (**27**)	*E. fraudatrix*	[29]
Cucumarioside H_2_ (**28**)	*E. fraudatrix*	[30]	Cucumarioside H_4_ (**29**)	*E. fraudatrix*	[30]
Cucumarioside H_5_ (**30**)	*E. fraudatrix*	[29]	Cucumarioside H_6_ (**31**)	*E. fraudatrix*	[29]
Cucumarioside H_7_ (**32**)	*E. fraudatrix*	[29]	Cucumarioside H_8_ (**33**)	*E. fraudatrix*	[29]
Cucumarioside I_1_ (**34**)	*E. fraudatrix*	[31]	Cucumarioside I_2_ (**35**)	*E. fraudatrix*	[32]
Cucumarioside I_3_ (**36**)	*E. fraudatrix*	[31]	Frondoside A (**37**)	*C. frondosa*	[33]
Frondoside B (**38**)	*C. frondosa*	[34]	Frondoside A_2_-1 (**39**)	*C. frondosa*	[35]
Frondoside A_2_-2 (**40**)	*C. frondosa*	[35]	Frondoside A_2_-3 (**41**)	*C. frondosa*	[35]
Frondoside A_2_-4 (**42**)	*C. frondosa*	[36]	Calcigeroside C_2_ (**43**)	*P. calcigera*	[37]
Calcigeroside D_2_ (**44**)	*P. calcigera*	[38]	Calcigeroside E (**45**)	*P. calcigera*	[38]
Colochiroside A (**46**)	*C. anceps*	[39]	Cucumarioside C_1_(**47**)	*E. fraudatrix*	[40]
Cucumarioside C_2_ (**48**)	*E. fraudatrix*	[40]	Synallactoside B_2_ (**49**)	*S. nozawai*	[16]
Synallactoside C (**50**)	*S. nozawai*	[16]	Synaptoside A (**51**)	*Synapta maculata*	[41]
Okhotoside A_2_-1 (**52**)	*C. okhotensis*	[42]	Frondoside A_7_-1 (**53**)	*C. frondosa*	[43]
Frondoside A_7_-2 (**54**)	*C. frondosa*	[43]			

**Table 3:** Name and producing species of glycosides with 3β-hydroxyholost-7(8)-ene and four sugar units.

Compound Name	Producing Species	Reference	Compound Name	Producing Species	Reference
Liouvilloside A (**55**)	*Staurocucumis liouvillei*	[44]	Liouvilloside A_1_ (**56**)	*S. liouviellei*	[45]
Liouvilloside A_2_ (**57**)	*S. liouvillei*	[45]	Liouvilloside A_3_ (**58**)	*S. liouvillei*	[45]
Liouvilloside A_5_ (**59**)	*S. liouvillei*	[46]	Liouvilloside B (**60**)	*S. liouvillei*	[44]
Liouvilloside B_1_ (**61**)	*S. liouvillei*	[45]	Liouvilloside B_2_ (**62**)	*S. liouvillei*	[45]
Violaceuside A (**63**)	*P. violeceus*	[47]	Violaceuside B (**64**)	*P. violeceus*	[47]
Violaceuside I (**65**)	*P. violeceus*	[48]	Violaceuside II (**66**)	*P. violeceus*	[48]
Violaceuside III (**67**)	*P. violeceus*	[48]	Intercedenside A (**68**)	*M. intercedens*	[49]
Intercedenside B (**67**)	*Mensamria intercedens*	[49]	Intercedenside C (**70**)	*M. intercedens*	[49]
Intercedenside D (**71**)	*M. intercedens*	[50]	Intercedenside E (**72**)	*M. intercedens*	[50]
Intercedenside F (**73**)	*M. intercedens*	[50]	Intercedenside G (**74**)	*M. intercedens*	[50]
Intercedenside H (**75**)	*M. intercedens*	[50]	Intercedenside I (**76**)	*M. intercedens*	[50]
Patagonicoside A (**77**)	*Psolus patagonicus*	[51]	Patagonicoside B (**78**)	*P. patagonicus*	[52]
Patagonicoside C (**79**)	*P. patagonicus*	[52]	Philinopside A (**80**)	*P. quadrangularis*	[53]
Philinopside B (**81**)	*Pentacta quadrangularis*	[53]	Philinopside E (**82**)	*P. quadrangularis*	[54]
Philinopside F (**83**)	*P. quadrangularis*	[54]	Mollisoside A (**84**)	*A. mollis*	[55]
Mollisoside B_2_ (**85**)	*Australostichopus mollis*	[55]	Eximisoside A (**86**)	*P. eximius*	[56]
Pseudostichoposide A (**87**)	*Pseudostichopus trachus*	[57]	Cucumarioside F_1_ (**88**)	*E. fraudatrix*	[58]
Cucumarioside F_2_ (**89**)	*E. fraudatrix*	[58]	Pseudocnoside A (**90**)	*P. leoninus*	[59]
Typicoside A_1_ (**91**)	*Actinocucumis typica*	[60]	Typicoside A_2_ (**92**)	*A. typica*	[60]
Typicoside B_1_ (**93**)	*A. typica*	[60]	Typicoside C_1_ (**94**)	*A. typica*	[60]
Typicoside C_2_ (**95**)	*A. typica*	[60]	Frondoside A_1_ (**96**)	*C. okhotensis*	[61]
Okhotoside A_1_-1 (**97**)	*Cucumaria okhotensis*	[61]	Okhotoside B_1_ (**98**)	*C. okhotensis*	[62]
Okhotoside B_2_ (**99**)	*C. okhotensis*	[62]	Okhotoside B_3_ (**100**)	*C. okhotensis*	[62]
Colochiroside A_1_ (**101**)	*Colochirus robustus*	[63]	Colochiroside A_2_ (**102**)	*C. robustus*	[63]
Colochiroside A_3_ (**103**)	*C. robustus*	[63]	Colochiroside B_1_ (**104**)	*C. robustus*	[64]
Colochiroside B_2_ (**105**)	*C. robustus*	[64]	Colochiroside B_3_ (**106**)	*C. robustus*	[64]
Violaceusosides C (**107**)	*P. violaceus*	[65]	Violaceusosides D (**108**)	*P. violaceus*	[65]
Violaceusosides E (**109**)	*P. violaceus*	[65]	Violaceusosides G (**110**)	*P. violaceus*	[65]
Cucumarioside A_1_ (**111**)	*E. fraudatrix*	[66]	Cucumarioside A_2_ (**112**)	*E. fraudatrix*	[67]
Cucumarioside A_3_ (**113**)	*E. fraudatrix*	[66]	Cucumarioside A_4_ (**114**)	*E. fraudatrix*	[66]
Cucumarioside A_5_ (**115**)	*E. fraudatrix*	[66]	Cucumarioside A_6_ (**116**)	*E. fraudatrix*	[66]
Cucumarioside A_7_ (**117**)	*E. fraudatrix*	[67]	Cucumarioside A_11_ (**118**)	*E. fraudatrix*	[67]
Cucumarioside A_12_ (**119**)	*E. fraudatrix*	[66]	Cucumarioside A_13_ (**120**)	*E. fraudatrix*	[67]
Cucumarioside A_14_ (**121**)	*E. fraudatrix*	[67]	Cucumarioside A_15_ (**122**)	*E. fraudatrix*	[66]
Cucumarioside G_1_ (**123**)	*C. fraudatrix*	[68]	Cucumarioside G_3_ (**124**)	*E. fraudatrix*	[69]
Cucumarioside G_4_ (**125**)	*E. fraudatrix*	[70]	Pentactaside B (**126**)	*P. quadrangularis*	[71]
Pentactaside C (**127**)	*P. quadrangularis*	[71]	Pseudostichoposide B (**128**)	*P. trachus*	[72]
Variegatuside A (**129**)	*S. variegates*	[73]	Variegatuside C (**130**)	*S. variegates*	[21]
Synallactoside A_1_(**131**)	*S. nozawai*	[16]	Thelenotoside A (**132**)	*Thelenota ananas*	[74]
Thelenotoside B (**133**)	*T. ananas*	[74]	Cucumechinoside A (**134**)	*C. echinata*	[75]
Cucumechinoside B (**135**)	*C. echinata*	[75]	Cucumechinoside C (**136**)	*C. echinata*	[75]
Cucumechinoside D (**137**)	*C. echinata*	[75]	Cucumechinoside E (**138**)	*C. echinata*	[75]
Cucumechinoside F (**139**)	*C. echinata*	[75]	Lefevreoside A_1_ (**140**)	*C. lefevrei*	[76]
Lefevreoside A_2_ (**141**)	*C. lefevrei*	[76]	Lefevreoside C (**142**)	*C. lefevrei*	[76]
Lefevreoside D (**143**)	*C. lefevrei*	[76]			

**Table 4:** Name and producing species of glycosides with 3β-hydroxyholost-7(8)-ene and 1–3 sugar units.

Compound Name	Producing Species	Reference	Compound Name	Producing Species	Reference
Pentactaside I (**144**)	*Pentacta quadrangularis*	[77]	Pentactaside II (**145**)	*P. quadrangularis*	[77]
Cucumarioside B_1_ (**146**)	*E. fraudatrix*	[78]	Cucumarioside B_2_ (**147**)	*E. fraudatrix*	[78]
Pentactaside III (**148**)	*P. quadrangularis*	[77]	Stichoposide A (**149**)	*S. cloronotus*	[79]
Stichoposide B (**150**)	*Stichopus cloronotus*	[79]	Stichorrenoside A (**151**)	*Stichopus horrens*	[80]
Stichorrenoside B (**152**)	*S. horrens*	[80]	Stichorrenoside C (**153**)	*S. horrens*	[80]
Stichorrenoside D (**154**)	*S. horrens*	[80]	Hillaside A (**155**)	*H. hilla*	[81]

**Table 5:** Name and producing species of glycosides with 3β-hydroxyholost-9(11)-ene and six sugar units.

Compound Name	Producing Species	Reference	Compound Name	Pro. Species	Reference
Holotoxin A (**156**)	*Stichopus japonicus*	[82]	Holotoxin A_1_ (**157**)	*S. japonicus*	[22]
25,26-dihydroxyholotoxin A_1_ (**158**)	*Apostichopus japonicus*	[83]	Oxo-holotoxin A_1_ (**159**)	*A. japonicus*	[22]
Holotoxin B (**160**)	*S. japonicus*	[22]	Holotoxin B_1_ (**161**)	*S. japonicus*	[22]
Holotoxin D (**162**)	*S. japonicus*	[22]	Holotoxin D_1_ (**163**)	*A. japonicus*	[83]
Parvimoside A (**164**)	*Stichopus parvimensis*	[84]	Parvimoside B (**165**)	*S. parvimensis*	[84]
Bivittoside C (**166**)	*Bohadschia bivittata*	[85]	Bivittoside D (**167**)	*B. bivittata*	[85]
25-acetoxybivittoside D (**168**)	*Bohadschia marmorata*	[86]	Arguside B (**169**)	*B. argus*	[87]
Arguside C (**170**)	*B. argus*	[87]	Marmoratoside A (**171**)	*B. marmorata*	[86]
Marmoratoside B (**172**)	*B. marmorata*	[86]	Impatienside A (**173**)	*H. impatiens*	[86]
17α-hydroxyimpatienside A (**174**)	*B. marmorata*	[86]	Lessonioside A (**175**)	*H. lessoni*	[88]
Lessonioside B (**176**)	*Holothuria lessoni*	[88]	Lessonioside D (**177**)	*H. lessoni*	[88]
Variegatuside E (**178**)	*S. variegates*	[21]	Lessonioside C (**179**)	*H. lessoni*	[88]
Lessonioside E (**180**)	*Holothuria lessoni*	[88]	Lessonioside F (**181**)	*H. lessoni*	[88]
Lessonioside G (**182**)	*H. lessoni*	[88]	Holothurinoside F (**183**)	*B. subrubra*	[89]
Holothurinoside H (**184**)	*B. subrubra*	[89]	Holothurinoside H_1_ (**185**)	*B. subrubra*	[89]
Holothurinoside I (**186**)	*B. subrubra*	[89]	Holothurinoside I_1_ (**187**)	*B. subrubra*	[89]
Holothurinoside K_1_ (**188**)	*B. subrubra*	[89]	Cladoloside C (**189**)	*C. schmeltzii*	[90]
Cladoloside C_1_ (**190**)	*Cladolabes schmeltzii*	[90]	Cladoloside C_2_ (**191**)	*C. schmeltzii*	[90]
Cladoloside C_3_ (**192**)	*C. schmeltzii*	[91]	Cladoloside D (**193**)	*C. schmeltzii*	[90]
Cladoloside G (**194**)	*C. schmeltzii*	[91]	Cladoloside H_1_ (**195**)	*C. schmeltzii*	[91]
Cladoloside H_2_ (**196**)	*C. schmeltzii*	[91]	Cladoloside K_1_ (**197**)	*C. schmeltzii*	[92]
Cladoloside K_2_ (**198**)	*C. schmeltzii*	[92]	Cladoloside L_1_ (**199**)	*C. schmeltzii*	[92]

**Table 6:** Name and producing species of glycosides with 3β-hydroxyholost-9(11)-ene and five sugar units.

Compound Name	Producing Species	Reference	Compound Name	Producing Species	Reference
Holothurinoside A (**200**)	*Holothuria forskalii*	[93]	17-dehydroxyholothurinoside A (**201**)	*Holothuria* *grisea*	[94]
Holothurinoside A_1_ (**202**)	*H.lessoni*	[95]	Holothurinoside B (**203**)	*H. forskalii*	[93]
Kolgaoside A (**204**)	*Kolga hyalina*	[96]	Kolgaoside B (**205**)	*K. hyalina*	[96]
Griseaside A (**206**)	*H. grisea*	[94]	Impatienside B (**207**)	*H. axiloga*	[97]
Arguside F (**208**)	*Holothuria* *axiloga*	[97]	Pervicoside D (**209**)	*H. axiloga*	[97]
Cladoloside B (**210**)	*A. japonicus*	[22]	Cladoloside B_1_ (**211**)	*C. schmeltzii*	[90]
Cladoloside B_2_ (**212**)	*C. schmeltzii*	[90]	Holothurinoside E (**213**)	*H. lessoni*	[95]
Holothurinoside E_1_ (**214**)	*H. lessoni*	[95]	Holothurinoside M (**215**)	*H. lessoni*	[95]
Achlioniceoside A_1_ (**216**)	*A. violaecuspidata*	[98]	Achlioniceoside A_2_ (**217**)	*A. violaecuspidata*	[98]
Achlioniceoside A_3_ (**218**)	*A. violaecuspidata*	[98]	Ds-penaustroside C (**219**)	*P. australis*	[99]
Ds-penaustroside D (**220**)	*Pentacta australis*	[99]	Frondoside A_2_-6 (**221**)	*C. frondosa*	[35]
Cladoloside E_1_ (**222**)	*C. schmeltzii*	[91]	Cladoloside E_2_ (**223**)	*C. schmeltzii*	[91]
Cladoloside F_1_ (**224**)	*C. schmeltzii*	[91]	Cladoloside F_2_ (**225**)	*C. schmeltzii*	[91]
Cercodemasoide A (**226**)	*C. anceps*	[100]	Cladoloside I_1_ (**227**)	*C. schmeltzii*	[92]
Cladoloside I_2_ (**228**)	*C. schmeltzii*	[92]	Cladoloside J_1_ (**229**)	*C. schmeltzii*	[92]

**Table 7:** Name and producing species of glycosides with 3β-hydroxyholost-9(11)-ene and four sugar units.

Compound Name	Producing Species	Reference	Compound Name	Producing Species	Reference
Holothurin A (**230**)	*Actinopyga agassizi*	[101]	Holothurin A_1_ (**231**)	*H. grisea*	[102]
Holothurin A_3_ (**232**)	*Holothuria scabra*	[103]	Holothurin A_4_ (**233**)	*H. scabra*	[103]
Holothurinoside C (**234**)	*H. forskalii*	[93]	Scabraside A (**235**)	*H. scabra*	[104]
Scabraside B (**236**)	[104]	104]	Scabraside D (**237**)	*H. scabra*	[105]
Fuscocineroside A (**238**)	*H. fuscocinerea*	[106]	Fuscocineroside B (**239**)	*H. fuscocinerea*	[106]
17-hydroxy fuscocineroside B (**240**)	*B. marmorata*	[107]	25-hydroxy-fuscocineroside B (**241**)	*B. marmorata*	[107]
Fuscocineroside C (**242**)	*H. fuscocinerea*	[106]	Echinoside A (**243**)	*A. echinites*	[108]
Ds-echinoside A (**244**)	*P. graeffei*	[109]	24-dehydroechinoside A (**245**)	*H. scabra*	[105]
22-hydroxy-24-dehydroechinoside A (**246**)	*Actinopyga flammea*	[110]	24-hydroxy-25-dehydroechinoside A (**247**)	*A. flammea*	[110]
25-hydroxydehydroechinoside A (**248**)	*A. flammea*	[110]	22-acetoxyechinoside A (**249**)	*A. flammea*	[110]
Desholothurin A (**250**)	*P. graeffei*	[93]	Pervicoside A (**251**)	*H. pervicax*	[111]
Pervicoside B (**252**)	*H. pervicax*	[111]	Pervicoside C (**253**)	*H. pervicax*	[111]
Arguside A (**254**)	*Bohadschia argus*	[112]	Holothurinoside J_1_ (**255**)	*P. graeffei*	[95]
Hemoiedemoside A (**256**)	*H. spectabilis*	[113]	Hemoiedemoside B (**257**)	*H. spectabilis*	[113]
Arguside D (**258**)	*B. argus*	[114]	Arguside E (**259**)	*B. argus*	[114]
Psolusoside A (**260**)	*Psolus fabricii*	[115]	Liouvilloside A_4_ (**261**)	*S. liouvillei*	[46]
Bivittoside B (**262**)	*Bohadschia bivitta*	[85]	Holothurinoside X (**263**)	*H. lessoni*	[116]
Holothurinoside Y (**264**)	*H.lessoni*	[116]	Holothurinoside Z (**265**)	*H. lessoni*	[116]
Cladoloside A_1_ (**266**)	*Cladolabes chmeltzii*	[117]	Cladoloside A_2_ (**267**)	*C. chmeltzii*	[117]
Cladoloside A_3_ (**268**)	*C. chmeltzii*	[117]	Cladoloside A_4_ (**269**)	*C. chmeltzii*	[117]
Cladoloside A_5_ (**270**)	*C. chmeltzii*	[117]	Cladoloside A_6_ (**271**)	*C. chmeltzii*	[117]
Colochiroside C (**272**)	*C. chmeltzii*	[64]	Colochiroside D (**273**)	*C. robustus*	[63]
Mollisoside B_1_ (**274**)	*A. mollis*	[55]	Neothyonidioside (**275**)	*A. mollis*	[118]

**Table 8:** Name and producing species of glycosides with 3β-hydroxyholost-9(11)-ene and 1–3sugar units.

Compound Name	Producing Species	Reference	Compound Name	Producing Species	Reference
Cercodemasoide B (**276**)	*Cercodemas anceps*	[100]	Cercodemasoide C (**277**)	*C. anceps*	[100]
Cercodemasoide D (**278**)	*C. anceps*	[100]	Cercodemasoide E (**279**)	*C. anceps*	[100]
Holothurin B (**280**)	*Holothuria lessoni*	[119]	Holothurin B_1_ (**281**)	*H. lessoni*	[120]
Holothurin B_2_ (**282**)	*H. polii*	[121]	Holothurin B_3_ (**283**)	*H. polii*	[121]
Holothurin B_4_ (**284**)	*H. polii*	[121]	Holothurinoside D (**285**)	*H. forskalii*	[93]
Leucospilotaside A (**286**)	*H. leucospilota*	[122]	Leucospilotaside B (**287**)	*H. leucospilota*	[122]
Bivittoside A (**288**)	*Bohadschia bivittata*	[85]	Echinoside B (**289**)	*A. echinites*	[108]
24-dehydroechinoside B (**290**)	*Actinopyga mauritiana*	[123]	Hillaside C (**291**)	*Holothuria hilla*	[124]
Hillaside B (**292**)	*H. hilla*	[81]			

**Table 9:** Name and producing species of holostane glycosides with 3β-hydroxyholost-8(9)-ene skeleton.

Compound Name	Producing Species	Reference	Compound Name	Producing Species	Reference
Synaptoside A_1_ (**293**)	*Synapta maculata*	[41]	Variegatuside B (**294**)	*Stichopus variegates*	[73]
Variegatuside D (**295**)	*Stichopus variegates*	[21]			

**Table 10:** Name and producing species of nonholostane glycosides.

Compound Name	Producing Species	Reference	Compound Name	Producing Species	Reference
Cucumarioside G_2_ (**296**)	*E. fraudatrix*	[125]	Cucumarioside A_10_ (**297**)	*E. fraudatrix*	[67]
Calcigeroside B (**298**)	*P. calcigera*	[37]	Calcigeroside C_1_ (**299**)	*P. calcigera*	[37]
Cucumarioside H_3_ (**300**)	*E. fraudatrix*	[30]	Cucumarioside A_3_-2 (**301**)	*C. conicospermium*	[26]
Cucumarioside A_3_-3 (**302**)	*C. conicospermium*	[26]	Koreoside A (**303**)	*C. koraiensis*	[126]
Isokoreoside A (**304**)	*C. conicospermium*	[26]	Cucumarioside A_8_ (**305**)	*E. fraudatrix*	[67]
Cucumarioside A_9_ (**306**)	*E. fraudatrix*	[67]	Holotoxin F (**307**)	*A. japonicus*	[22]
Holotoxin G (**308**)	*A. japonicus*	[22]	Holotoxin H (**309**)	*S. japonicus*	[127]
Holotoxin I (**310**)	*S. japonicus*	[127]	Ds-penaustroside A **(311**)	*P. australis*	[99]
Ds-penaustroside B (**312**)	*P. australis*	[99]	Frondoside C (**313**)	*C. frondosa*	[128]
Psolusoside B (**314**)	*Psolus fabricii*	[129]	Kuriloside A (**315**)	*D. kurilensi*	[130]
Kuriloside C (**316**)	*D. kurilensi*	[130]	Frondoside A_2_-7 (**317**)	*C. frondosa*	[36]
Frondoside A_2_-8 (**318**)	*C. frondosa*	[36]	Frondoside A_7_-3 (**319**)	*C. frondosa*	[43]
Frondoside A_7_-4 (**320**)	*C. frondosa*	[43]	Isofrondoside C (**321**)	*C. frondosa*	[43]
Fallaxoside B_1_ (**322**)	*C. fallax*	[131]	Fallaxoside C_1_ (**323**)	*C. fallax*	[132]
Fallaxoside C_2_ (**324**)	*C. fallax*	[132]	Fallaxoside D_1_ (**325**)	*C. fallax*	[132]
Fallaxoside D_2_ (**326**)	*C. fallax*	[132]	Fallaxoside D_3_ (**327**)	*C. fallax*	[131]
Fallaxoside D_4_ (**328**)	*C. fallax*	[133]	Fallaxoside D_5_ (**329**)	*C. fallax*	[133]
Fallaxoside D_6_ (**330**)	*C. fallax*	[133]	Fallaxoside D_7_ (**331**)	*C. fallax*	[133]
Magnumoside A_1_ (**332**)	*Massinium magnum*	[134]	Magnumoside A_2_ (**333**)	*M. magnum*	[134]
Magnumoside A_3_ (**334**)	*M. magnum*	[134]	Magnumoside A_4_ (**335**)	*M. magnum*	[134]
Magnumoside B_1_ (**336**)	*M. magnum*	[134]	Magnumoside B_2_ (**337**)	*M. magnum*	[134]
Magnumoside C_1_ (**338**)	*M. magnum*	[134]	Magnumoside C_2_ (**339**)	*M. magnum*	[134]
Magnumoside C_4_ (**340**)	*M. magnum*	[134]	Colochiroside E (**341**)	*C. robustus*	[135]

**Table 11:** Remarkable biological activities exhibited by some sea cucumber glycosides.

Compound	Activity	Against/For	Activity Result	Reference
Hillaside C (**285**)	Cytotoxic	Human tumor cell lines	IC_50_: 0.15–3.20 µg/mL	[124]
Hemoiedemoside A (**251**)	Antifungal	*C. cucumerinum*	20 µg/disc: 23 mm zone	[113]
Fuscocineroside C (**237**)	Cytotoxic	Human tumor cell lines	IC_50_: 0.88 and 0.58 µg/mL	[106]
Intercedenside A (**66**)	Cytotoxic	Human tumor cell lines	ED_50_: 0.96–4.0 µg/mL	[49]
Intercedenside B (**67**)	Cytotoxic	Human tumor cell lines	ED_50_: 0.61–2.0 µg/mL	[49]
Intercedenside C (**68**)	Cytotoxic	Human tumor cell lines	ED_50_: 0.96–4.0 µg/mL	[49]
Holothurinoside A (**195**)	Cytotoxic	Human tumor cell lines	IC_50_: 0.33–0.71 µg/mL	[93]
Holothurinoside C (**229**)	Cytotoxic	Human tumor cell lines	IC_50_: 0.16–0.93 µg/mL	[93]
Liouvilloside A (**53**)	Virucidal	Herpes simplex virus	<10 µg/mL	[44]
Leucospilotaside B (**281**)	Cytotoxic	Human tumor cell lines	IC_50_: 0.44–2.62 µg/mL	[122]
Arguside B (**164**)	Cytotoxic	Human tumor cell lines	IC_50_: 0.38–2.60 µg/mL	[87]
Arguside C (**165**)	Cytotoxic	Human tumor cell lines	IC_50_: 0.38–2.60 µg/mL	[87]
Philinopside A (**78**)	Cytotoxic	Human tumor cell lines	IC_50_: 1.70–3.50 µg/mL	[52]
Philinopside B (**79**)	Cytotoxic	Human tumor cell lines	IC_50_: 0.75–3.0 µg/mL	[53]
Cucumarioside A_2_-2 (**18**)	Hemolytic	Erythrocyte of mouse	ED50: 0.87 at 10^−6^ M	[136]
Holothurin B (**274**)	Antifungal	*T. mentagrophytes*	MIC-1.5 µg/mL	[137]
Holothurin A_3_ (**227**)	Cytotoxic	Human tumor cell lines	IC_50_ = 0.32–0.87 μg/mL	[103]
Holothurin A_4_ (**228**)	Cytotoxic	Human tumor cell lines	IC_50_ = 0.57–1.12 μg/mL	[103]
Scabraside A (**230**)	Antifungal	Eight pathogenic fungal strains	MIC_80_: 2–8 μg/mL	[138]
Echinoside A (**238**)	Antifungal	Eight pathogenic fungal strains	MIC_80_: 1–8 μg/mL	[108]
Cucumarioside A_2_-2 (**18**)	immunomodulatory	Increased lysosomal activity	0.2–20 ng/mouse	[139]
Frondoside A (**37**)	immunomodulatory	Enhanced phagocytosis	0.001 μg/mL	[140]
Philinopside E (**80**)	Cytotoxicity	Ten tumor cell lines	ED_50_: 0.75–3.50 μg/mL	[54]
Holotoxin A_1_ (**152**)	Antifungal	Five pathogenic fungi	MIC: 0.5–1.0 µg/mL	[22]
Cucumarioside A_1_ (**106**)	Hemolytic	Mouse erythrocytes	MEC_100_: 0.7 ± 0.1 µg/mL	[66]
